# Total copy number variation as a prognostic factor in adult astrocytoma subtypes

**DOI:** 10.1186/s40478-019-0746-y

**Published:** 2019-06-10

**Authors:** Kanish Mirchia, Adwait Amod Sathe, Jamie M. Walker, Yelena Fudym, Kristyn Galbraith, Mariano S. Viapiano, Robert J. Corona, Matija Snuderl, Chao Xing, Kimmo J. Hatanpaa, Timothy E. Richardson

**Affiliations:** 1 Department of Pathology, State University of New York, Upstate Medical University, Syracuse, NY 13210 USA; 2 Eugene McDermott Center for Human Growth & Development, University of Texas Southwestern Medical Center, Dallas, TX 75390 USA; 3 Department of Pathology, University of Texas Health Science Center, San Antonio, TX 78229 USA; 4 Glenn Biggs Institute for Alzheimer’s & Neurodegenerative Diseases, University of Texas Health Science Center, San Antonio, TX 78229 USA; 5 Department of Neuroscience and Physiology, State University of New York, Upstate Medical University, Syracuse, NY 13210 USA; 6 Department of Neurosurgery, State University of New York, Upstate Medical University, Syracuse, NY 13210 USA; 7 Department of Pathology, New York University Langone Medical Center, New York City, NY 10016 USA; 8 Department of Bioinformatics, University of Texas Southwestern Medical Center, Dallas, TX 75390 USA; 9 Department of Population and Data Sciences, University of Texas Southwestern Medical Center, Dallas, TX 75390 USA; 10 Department of Pathology, University of Texas Southwestern Medical Center, Dallas, TX 75390 USA

**Keywords:** Copy number variation, CNV, Astrocytoma, Glioma, Glioblastoma, GBM, TCGA

## Abstract

Since the discovery that *IDH1/2* mutations confer a significantly better prognosis in astrocytomas, much work has been done to identify other molecular signatures to help further stratify lower-grade astrocytomas and glioblastomas, with the goal of accurately predicting clinical outcome and identifying potentially targetable mutations. In the present study, we subclassify 135 astrocytomas (67 *IDH*-wildtype and 68 *IDH*-mutant) from The Cancer Genome Atlas dataset (TCGA) on the basis of grade, *IDH*-status, and the previously established prognostic factors, *CDK4* amplification and *CDKN2A/B* deletion, within the *IDH*-mutant groups. We analyzed these groups for total copy number variation (CNV), total mutation burden, chromothripsis, specific mutations, and amplifications/deletions of specific genes/chromosomal regions. Herein, we demonstrate that across all of these tumor groups, total CNV level is a relatively consistent prognostic factor. We also identified a trend towards increased levels of chromothripsis in tumors with lower progression-free survival (PFS) and overall survival (OS) intervals. While no significant differences were identified in overall mutation load, we did identify a significantly higher number of cases with mutations in genes with functions related to maintaining genomic stability in groups with higher mean CNV and worse PFS and OS intervals, particularly in the *IDH*-mutant groups. Our data further support the case for total CNV level as a potential prognostic factor in astrocytomas, and suggest mutations in genes responsible for overall genomic instability as a possible underlying mechanism for some astrocytomas with poor clinical outcome.

**Electronic supplementary material:**

The online version of this article (10.1186/s40478-019-0746-y) contains supplementary material, which is available to authorized users.

## Introduction

Diffuse gliomas are among the most common primary CNS tumors, representing approximately 27% of all primary brain tumors [29, 30]. Due to their infiltrative nature, these tumors are surgically incurable, although the exact prognosis depends on numerous histologic and molecular factors. The standard of care now dictates molecular classification of gliomas based on *IDH1/2* mutation status as *IDH*-mutant gliomas have a significantly better prognosis than their *IDH*-wildtype grade-matched counterparts [25]. While histologic grade shows correlation with overall survival within these molecular groups, there remains significant heterogeneity in clinical outcome.

Since the widespread adoption of the 2016 WHO classification system, much work has been done to find further molecular markers to sub-stratify both *IDH*-mutant and *IDH*-wildtype astrocytomas in hopes of better predicting tumor behavior and outcome, including identification of secondary mutations, focal genetic alterations, methylation patterns, and multivariate prognostic models [3, 24, 42, 44]. Within the *IDH*-wildtype groups, these studies have suggested that lower-grade gliomas (LGG) with *EGFR* amplification, gain of chromosome 7 and loss of 10, or *TERT* promoter mutations will have aggressive clinical courses and outcomes similar to *IDH*-wildtype glioblastoma, regardless of histologic features. In *IDH*-mutant groups, lower-grade tumors with alterations in genes in the retinoblastoma pathway, including amplification of *CDK4* and deletion of *CDKN2A/B*, demonstrate significantly worse clinical behavior and shorter patient survival [1, 5, 8, 33].

Previous work has demonstrated that *IDH*-mutant glioblastomas have higher levels of total copy number variation (CNV) across the entire genome and evidence of more frequent chromothripsis than lower-grade*IDH*-mutant astrocytomas [9]. We subsequently showed that in *IDH*-mutant grade II and III astrocytomas, this increased level of CNV was present before progression to glioblastoma in cases with exceptionally poor outcomes, defined by rapid progression to glioblastoma and short survival times after initial diagnosis [36, 37]. The poor outcome appeared to be directly correlated with overall CNV, but not other factors, including mutation burden or differences in methylation profiles, suggesting that this large scale CNV pattern could potentially override the beneficial effect of *IDH*-mutant status.

To better understand the effect of CNV, we analyzed 135 astrocytic tumors from The Cancer Genome Atlas (TCGA) (67 *IDH*-wildtype and 68 *IDH*-mutant cases) with respect to clinical outcome, CNV levels, chromosomal and specific gene amplification and deletion events, chromothripsis, total mutation load, specific mutations in known glioma/GBM genes, and mutations in genes associated with overall genomic instability. Building on our previous results, we performed wide scale genomic analysis, on a framework of pre-established prognostic factors including grade, *IDH1/2*-status, and the presence of *CDK4* amplifications or *CDKN2A/B* deletions. With the exception of 2 *IDH1/2*-wildtype cases, *CDK4* amplification and *CDKN2A/B* deletion were found to be mutually exclusive. We divided the cases into 5 groups: *IDH1/2*-mutant LGG without *CDK4* amplification or *CDKN2A/B* deletion (Group 1), *IDH1/2*-mutant LGG with either *CDK4* amplification or *CDKN2A/B* deletion LGG (Group 2), *IDH1/2*-mutant GBM (Group 3), *IDH1/2*-wildtype LGG (Group 4), and *IDH1/2*-wildtype GBM (Group 5).

We demonstrate that higher levels of CNV and chromothripsis are correlated with clinical outcome in the *IDH*-mutant groups, while the *IDH*-wildtype groups had uniformly high CNV levels and poor outcomes. Other prognostic factors appear to be inconsistent. We also identified a significantly higher number of mutations in genes involved with overall genomic stability, paralleling levels of overall CNV and chromothripsis, in the cases with worse prognosis. While defining the exact role of genes involved in progression may still be needed for development of individualized targeted therapies, use of CNV could potentially serve as a clinically impactful model for prognostication of different astrocytoma subtypes, and may aid in our understanding of the underlying biology of these tumor types.

## Methods

### TCGA case selection

Using the cBioportal interface, we performed a search of 380 glioblastoma cases and 539 lower-grade gliomas (LGG, defined here as WHO grade II-III) [6, 9, 14]. The original histologic diagnoses reported in TCGA included astrocytoma, oligoastrocytoma, anaplastic astrocytoma, anaplastic oligoastrocytoma, and glioblastoma. All cases were manually reclassified according the WHO 2016 criteria as diffuse astrocytomas (WHO grade II-IV) by histology, intact 1p/19q status, and *IDH1/2*, *ATRX*, and *TP53* status. Oligodendrogliomas were specifically excluded on the basis of 1p/19q co-deletion, as these tumors have been shown to have different underlying molecular drivers and a more favorable clinical outcome as a group. All cases selected represented the first resection specimen and were segregated into lower-grade (WHO grades II and III) [9, 28, 35] and GBM (WHO grade IV) within the *IDH1/2* mutation groups. We identified 5 groups based on previously identified prognostic factors, including histologic grade, *IDH1/2*, *CDK4*, and *CDKN2A/B* status [1, 8, 25, 31, 45] and selected groups of TCGA cases that met these criteria: Group 1, *IDH1/2*-mutant LGG without *CDK4* amplification or *CDKN2A/B* deletion (*n* = 24, mean age = 38.8 ± 1.9 years); Group 2, *IDH1/2*-mutant, *CDK4*-amplified/*CDKN2A/B*-deleted LGG (*n* = 22, mean age = 38.8 ± 1.9 years); Group 3, *IDH1/2*-mutant GBM (*n* = 22, mean age = 40.5 ± 2.7 years); Group 4, *IDH1/2*-wildtype LGG (*n* = 25, mean age = 54.0 ± 2.6 years); Group 5, *IDH1/2*-wildtype GBM (*n* = 42, mean age = 62.8 ± 1.7 years) (Table 1).

**Table 1:** Summary of available clinical, histologic, and molecular data from each astrocytoma subgroup analyzed

Group	Tumor Type	*n*	Age at Onset (years)	Median Progression-Free Survival (months)	Median Overall Survival (months)	Histologic Grade (II/III/IV)	CNV Level (%)	Cases with Chromothripsis	Mutation Count	Instability Gene Mutations
1	*IDH*-mut LGG	24	38.8 ± 1.9	95	> 172	12/12/0	9.1 ± 1.6	2 (8.3%)	43 ± 10.5	1 (4.1%)
2	*IDH*-mut *CDK4*/									
	*CDKN2A/B* LGG	22	38.8 ± 1.9	32	36	4/18/0	21.3 ± 2.5	6 (27.3%)	33.3 ± 1.3	7 (31.8%)
3	*IDH*-mut GBM	22	40.5 ± 2.7	10	33	0/0/22	20 ± 2.7	9 (40.9%)	67.4 ± 2.75	8 (36.4%)
4	*IDH*-wt LGG	25	54.0 ± 2.6	10.5	15.5	0/25/0	19.9 ± 1.8	5 (20.0%)	64.9 ± 16.7	5 (20.0%)
5	*IDH*-wt GBM	42	62.8 ± 1.7	6	13	0/0/42	22.2 ± 1.6	11 (26.2%)	57.0 ± 2.5	10 (23.8%)

### Genetic and epigenetic analysis

The gene expression (Illumina HiSeq, RNASeq) and DNA methylation data (Illumina Human Methylation 450) was downloaded for the selected TCGA cases and analyzed with TCGAbiolinks [10]. The Affymetrix SNP 6.0 microarray data normalized to germline for copy number analysis for the same TCGA cases was downloaded from Broad GDAC Firehose (http://gdac.broadinstitute.org/runs/stddata__2016_01_28/). The fraction of copy number alterations was calculated from the above data as the fraction of the genome with log2 of copy number > 0.3 following the procedure used in cBioportal [14]. The mutation load is the number of nonsynonymous mutations seen in a sample. The differential analysis and visualization of mutations was done using Maftools [26]. The Ideogram for visualization of genome-wide copy number variation results was generated using Genome Decoration Page (https://www.ncbi.nlm.nih.gov/genome/tools/gdp). The pathway and network analyses were conducted using Qiagen’s IPA tool (www.qiagen.com/ingenuity) and R 3.4.1 (http://www.R-project.org/).

### GISTIC analysis

The GISTIC (Genomic Identification of Significant Targets in Cancer) 2.0 algorithm was used to identify regions of the genome that are significantly amplified or deleted between the 5 groups of *IDH1/2*-mutant and wildtype astrocytoma cases [27]. Each area of CNV is assigned a G-score that considers the amplitude of the alteration as well as the frequency of its occurrence across samples. The false discovery rate (FDR) was then used to determine the relative significance of each abnormality. Each region predicted to be significantly different between the 5 groups was screened for tumor suppressor genes, oncogenes, and other genes associated with glioma and malignancy [2, 27]. GISTIC 2.0 analysis was run using GenePattern [32].

### Mutation analysis of genes involved in maintenance of genomic stability

A group of genes with previously identified roles in cell proliferation and maintaining chromosomal stability were identified by a literature review and included the following genes: *APC*, *ATM*, *ATR*, *BLM*, *BRCA1* (*FANCS*), *BRCA2* (*FANCD1*), *BUB1B*, *CHK1*, *CLSPN*, *DNA-PK* (*PRKDC*), *EME1*, *FANCA*, *FANCB*, *FANCC*, *FANCD2*, *FANCE*, *FANCF*, *FANCG*, *FANCI*, *FANCJ* (*BRIP1*), *FANCL*, *FANCM*, *FANCN* (*PALB2*), *FANCO* (*RAD51C*), *FANCP* (*SLX4*), *FANCQ*, *FANCR*, *FANCT* (*UBE2T*), *HUS1*, *LIG4*, *MUS81*, *NBN*, *POLK*, *POLN*, *RAD51*, *RAD52*, *REV3*, *SMC1*, *SNM1B*, *TOP1*, *TP53*, *WRN*, and *XLF* [7, 16, 36]. Variant annotation was performed using COSMIC [13], dbSNP [39], ClinVar [22], CanProVar 2.0 [23], The 1000 Genomes Project [15], and FATHMM-MKL [40].

### Statistical analysis

Differences in patient age, mutation burden, and CNV were evaluated using Analysis of Variance (ANOVA). Significance of survival curves were calculated using the Mantel-Cox test (Log-rank test). Proportion of cases with chromothripsis and mutations specifically associated with genome instability were calculated using Fisher’s Exact test. Coefficients of variation (CNV vs survival times) were calculated using Pearson correlation coefficient. All statistical calculations were performed with GraphPad Prism version 7.04 (GraphPad, La Jolla, CA).

## Results

### Clinical characteristics

**Fig. 1:**
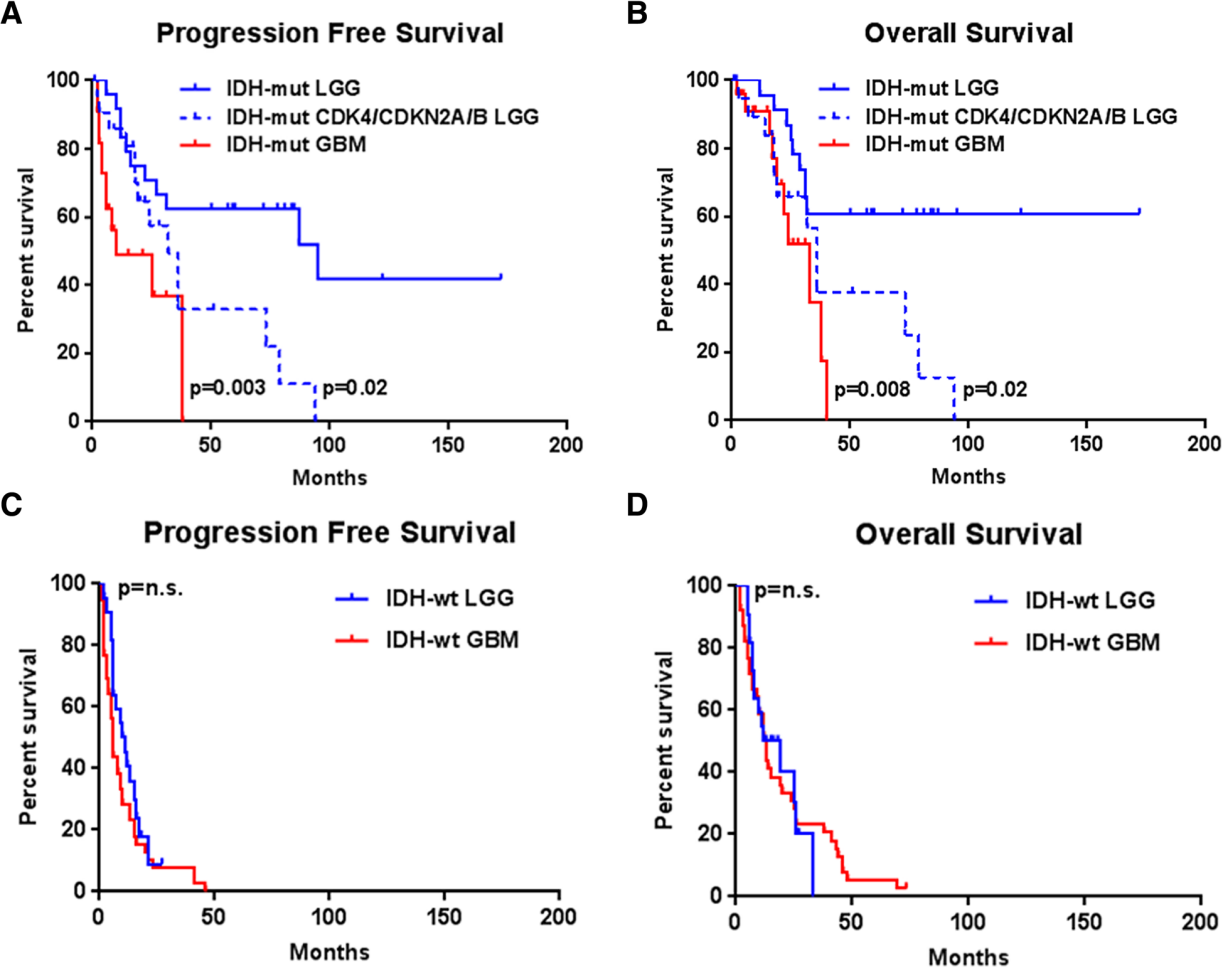
Kaplan-Meier survival curves demonstrating a significant difference between *IDH*-mutant LGGs without *CDK4* amplification or *CDKN2A/B* deletion and both *IDH*-mutant LGGs with *CDK4* or *CDKN2A/B* alterations (*p* = 0.0224) and *IDH*-mutant GBMs (*p* = 0.0032), but not between *IDH*-mutant LGGs with *CDK4* or *CDKN2A/B* alterations and *IDH*-mutant GBMs (*p* = 0.0769) in terms of progression-free survival (**a**). There was also a significant difference between *IDH*-mutant LGGs and both *IDH*-mutant LGGs with *CDK4* or *CDKN2A/B* alterations (*p* = 0.0150) and *IDH*-mutant GBMs (*p* = 0.0081), but not between *IDH*-mutant LGGs with *CDK4* or *CDKN2A/B* alterations and *IDH*-mutant GBMs (*p* = 0.2892) in terms of overall survival (**b**). No significant differences are identified between *IDH*-wildtype LGGs and *IDH*-wildtype GBMs in terms of progression-free survival (*p* = 0.2050) (**c**) or overall survival (*p* = 0.9351) (**d**)

**Fig. 2:**
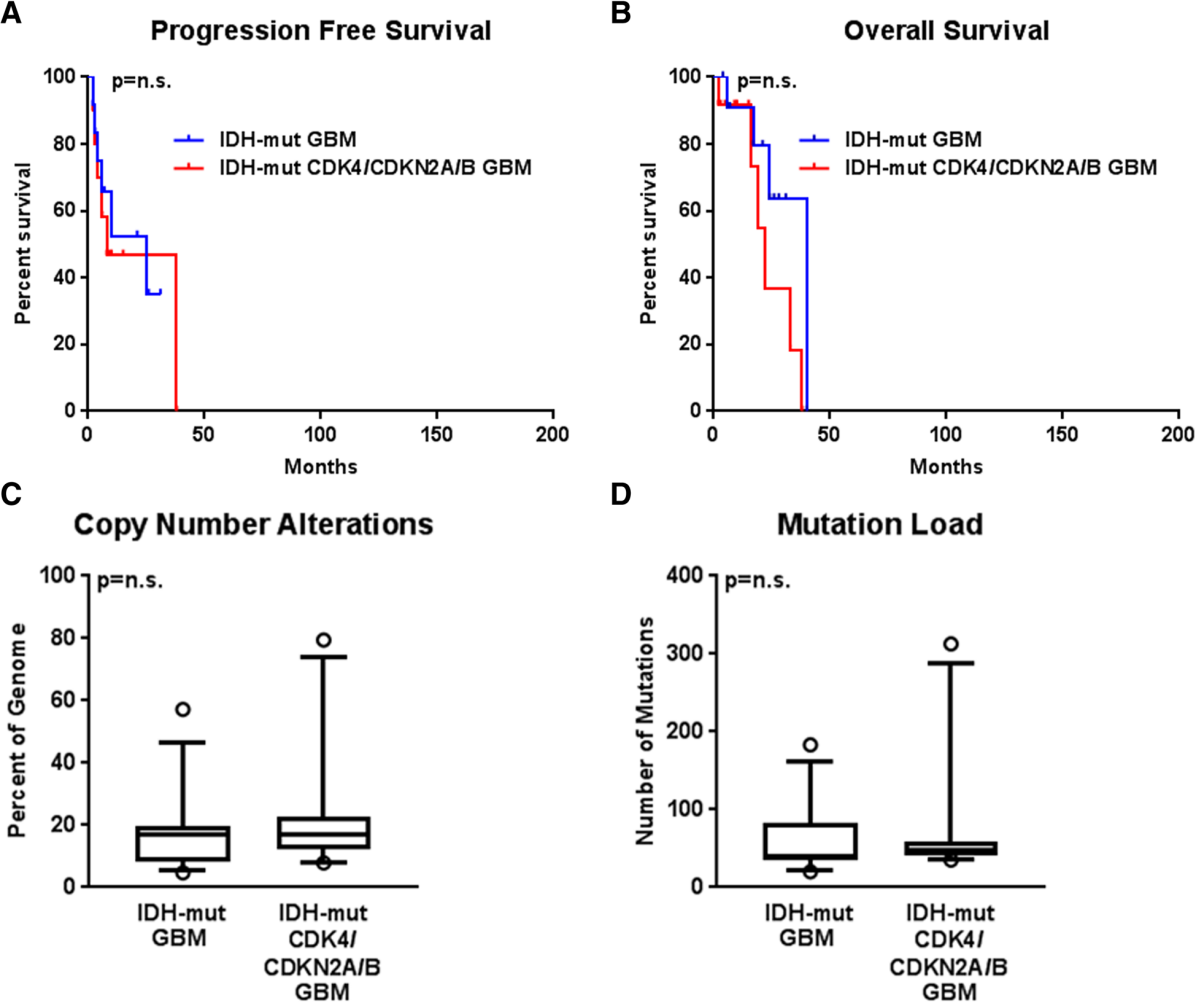
Comparison between *IDH*-mutant glioblastoma cases with and without amplifications of *CDK4* or deletions of *CDKN2A/B*. There is no significant difference in progression-free survival (*p* = 0.8406) (**a**), overall survival (*p* = 0.1471) (**b**), total copy number variation burden (*p* = 0.5326) (**c**), or total mutation burden (*p* = 0.6686) (**d**) between these groups

As previously demonstrated [1, 8], *IDH*-mutant LGGs (group 1) had a significantly longer progression-free survival (PFS; median 95 months) and overall survival (OS; > 172 months) than *IDH*-mutant LGGs with *CDK4* amplifications or *CDKN2A/B* deletions (group 2) (PFS 32 months, *p* = 0.0224; OS 36 months, *p* = 0.0150) and a significantly longer PFS and OS than *IDH*-mutant GBM (group 3) (PFS 10 months, *p* = 0.0032; OS 33 months, *p* = 0.0081). A significant difference was not found between *IDH*-mutant LGGs with *CDK4* amplifications or *CDKN2A/B* deletions (group 2) and *IDH*-mutant GBM (group 3) in terms of PFS (*p* = 0.0769) or OS (*p* = 0.2892) (Fig. 1a-b). No significant difference was found between *IDH*-wildtype LGGs (group 4) and *IDH*-wildtype GBM (group 5) in terms of PFS (*p* = 0.2050) or OS (*p* = 0.9351) (Fig. 1c-d). Amplifications in *CDK4* and deletions in *CDKN2A/B* did not have prognostic significance within the *IDH*-mutant GBM group in terms of PFS (*p* = 0.8406) or OS (*p* = 0.1471) (Fig. 2a-b).

No significant difference was identified in the median age of onset within the *IDH*-mutant groups 1–3, however there was a significant difference between the average age of onset in *IDH*-mutant LGG cases (38.8 ± 1.9 years) and *IDH*-wildtype LGG cases (54.0 ± 2.6 years) (*p* < 0.0001). There was also a significant difference in age of onset between *IDH*-wildtype LGGs (54.0 ± 2.6 years) and *IDH*-wildtype GBMs (62.8 ± 1.7 years) (*p* = 0.0047). There was a trend toward higher histologic tumor grade identified between groups 1 and 2. All *IDH1/2*-wildtype LGG tumors (group 4) were WHO grade III by histology at initial diagnosis (Table 1).

### Total copy number analysis differences

**Fig. 3:**
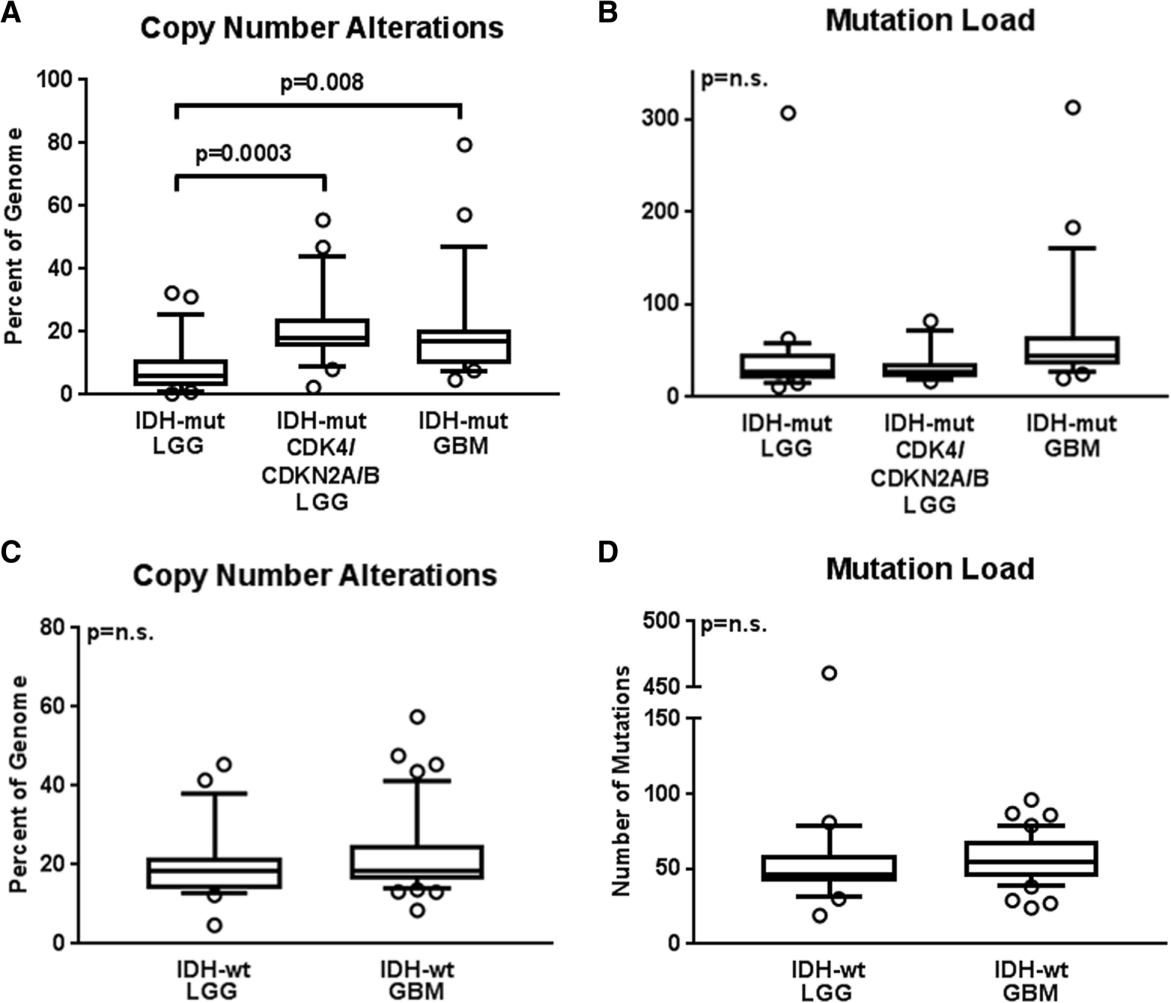
Total copy number variation averages demonstrating a significant difference between *IDH*-mutant LGGs without *CDK4* amplification or *CDKN2A/B* deletion and both *IDH*-mutant LGGs with *CDK4* or *CDKN2A/B* alterations (*p* = 0.0003) and *IDH*-mutant GBMs (*p* = 0.0078), but not between *IDH*-mutant LGGs with *CDK4* or *CDKN2A/B* alterations and *IDH*-mutant GBMs (*p* = 0.7783) (**a**); no significant difference was found in total mutation burden between any group of *IDH*-mutant astrocytoma (**b**). There was no significant difference between *IDH*-wildtype LGGs and *IDH*-wildtype GBMs in terms of overall copy number variation (*p* = 0.3732) (**c**) or total mutation burden (*p* = 0.5627) (**d**)

Mirroring the difference in clinical outcome, the total percentage of the genome with copy number alterations was low in the LGGs without *CDK4* or *CDKN2A/B* alterations and uniformly high in the other 4 groups (Table 1). Total copy number variation was 9.1 ± 1.6% in *IDH*-mutant LGGs (group 1), a significantly lower level than *IDH*-mutant LGGs with *CDK4* amplification or *CDKN2A/B* deletion (group 2) (21.3 ± 2.5%, *p* = 0.0003) or *IDH*-mutant GBM (group 3) (20.0 ± 2.7%, *p* = 0.0078). No significant difference was identified between any of the groups with statistically equivalent prognoses: group 2 vs group 3, *p* = 0.7758; group 3 vs group 5, *p* = 0.5277; or group 4 vs group 5, *p* = 0.3732) (Fig. 3a, c). No significant difference was noted when comparing *IDH*-mutant GBM cases with *CDK4* amplification or *CDKN2A/B* deletion to those without (*p* = 0.5326) (Fig. 2c). These calculations could not be meaningfully performed in either *IDH*-wildtype group due to the high frequency of *CDK4* and *CDKN2A/B* alterations.

**Fig. 4:**
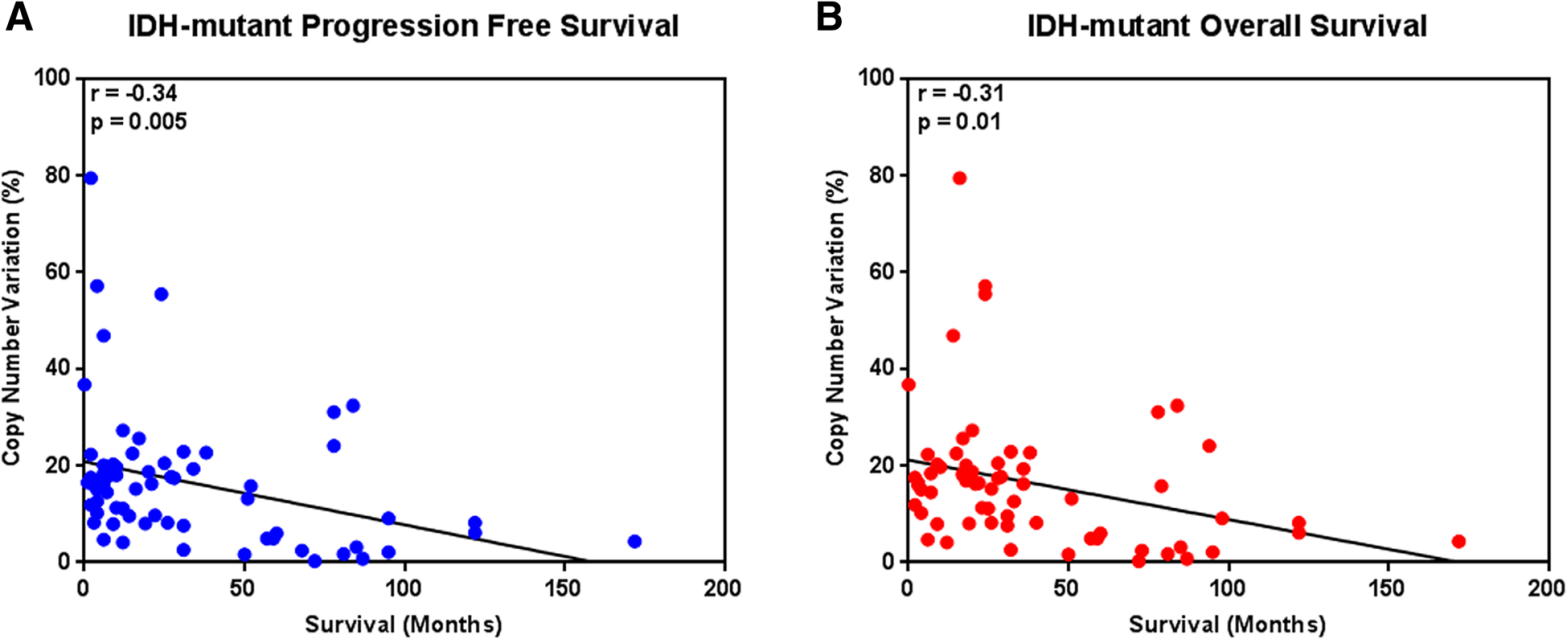
Scatter plots of copy number variation (%) plotted against survival time (months) in grouped *IDH*-mutant LGGs and *IDH*-mutant GBMs with Pearson’s R values, illustrating significant inverse correlations between the two data points in terms of (**a**) progression-free survival (*r* = − 0.3415; *p* = 0.0047) and (**b**) overall survival (*r* = − 0.3098; *p* = 0.0102)

In the *IDH*-mutant astrocytomas as a whole (groups 1–3), there was a statistically significant inverse correlation between the total copy number variation in each case and both the progression-free survival (*r* = − 0.3415; *p* = 0.0047) (Fig. 4a) and overall survival (*r* = − 0.3098; *p* = 0.0102) (Fig. 4b). Due to the uniformly high CNV level and poor prognosis in the *IDH*-wildtype tumor groups 4 and 5, no significant correlation was established between CNV and PFS or OS within these groups.

### Chromosomal analysis and GISTIC

**Fig. 5:**
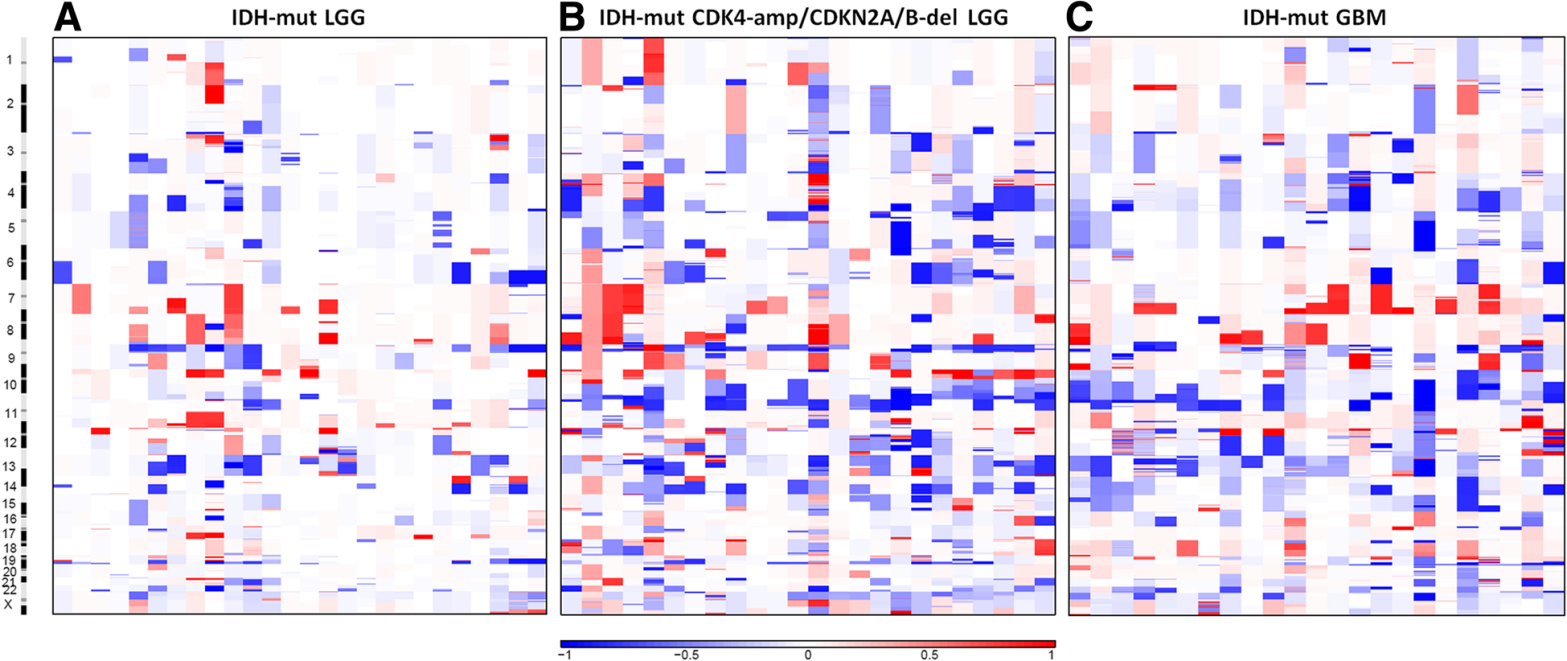
Overall amplification and deletion levels and chromosomal locations in *IDH*-mutant LGGs without *CDK4* amplification or *CDKN2A/B* deletion (**a**), IDH-mutant LGGs with either *CDK4* amplification or *CDKN2A/B* deletion (**b**), and IDH-mutant GBMs (**c**)

**Fig. 6:**
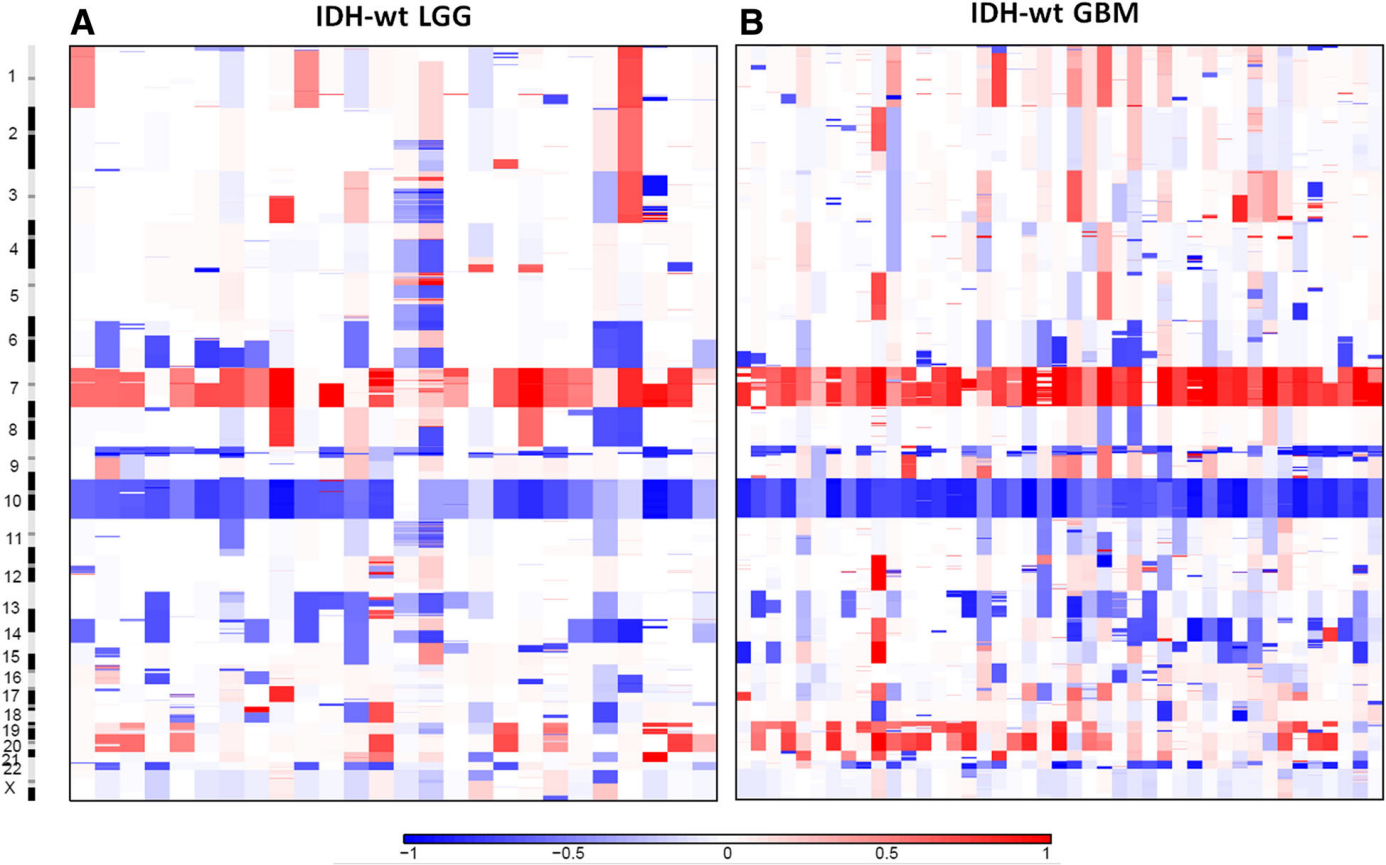
Overall amplification and deletion levels and chromosomal locations in *IDH*-wildtype LGGs (**a**) and *IDH*-wildtype GBMs (**b**)

Analysis of the *IDH*-mutant tumors (groups 1–3) revealed a heterogeneous assortment of genomic alterations with few consistent chromosomal regions with amplifications or deletions, although there is a clear increase in number of overall alterations between the group 1 *IDH*-mutant LGGs and the group 2 *IDH*-mutant LGGs with *CDK4* amplification/*CDKN2A/B* deletion and group 3 *IDH*-mutant GBM (Fig. 5), quantified in Fig. 3a. Conversely, *IDH*-wildtype LGGs and GBMs form a relatively homogeneous group with consistent amplifications, including large amplifications along chromosome 7, deletions on 9p, and deletions of chromosome 10 (Fig. 6).

**Fig. 7:**
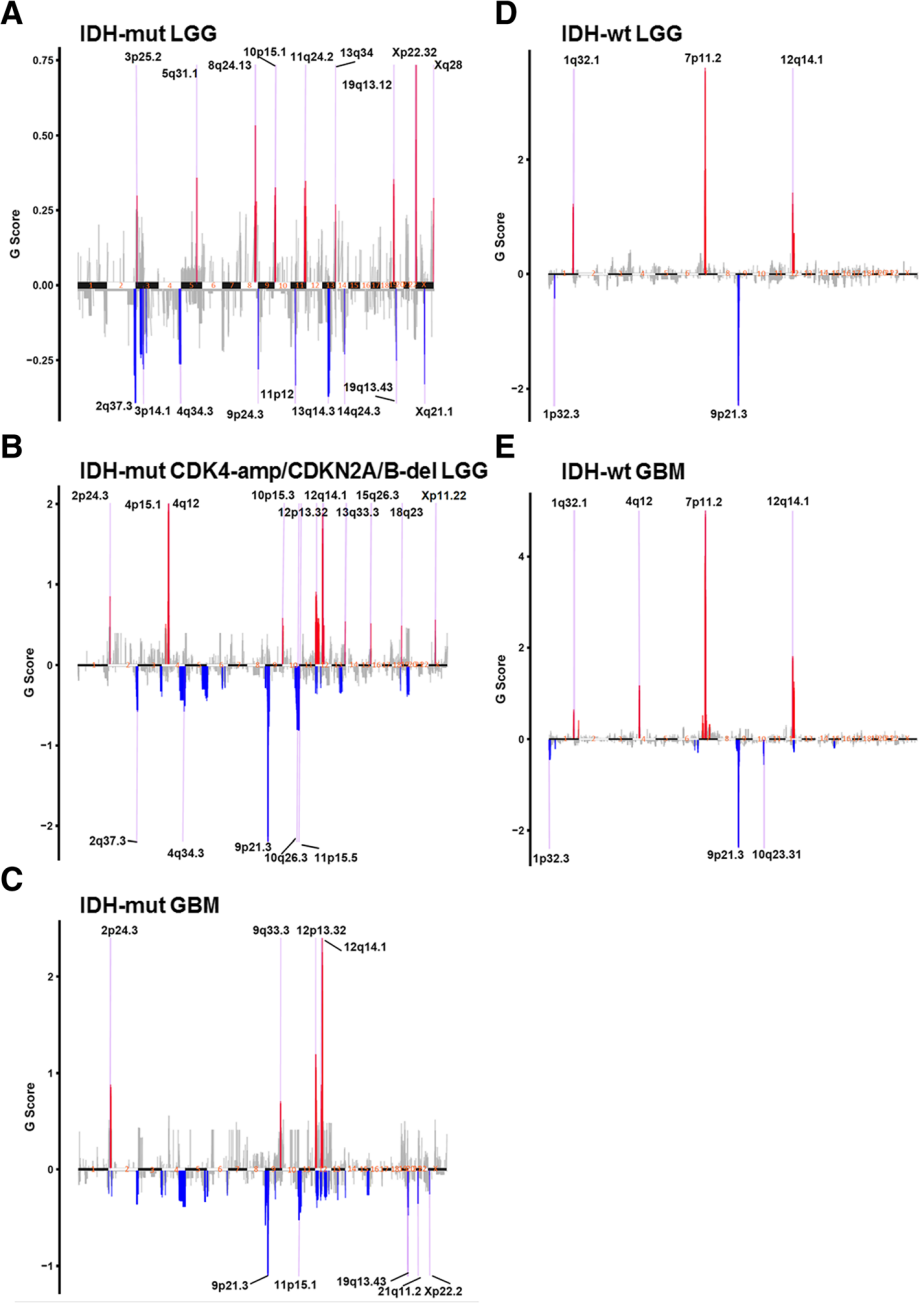
GISTIC analysis showing the most consistent and relevant cytoband alterations in *IDH*-mutant LGGs without *CDK4* amplification or *CDKN2A/B* deletion (**a**), *IDH*-mutant LGGs with either *CDK4* amplification or *CDKN2A/B* deletion (**b**), *IDH*-mutant GBMs (**c**), *IDH*-wildtype LGGs (**d**), and *IDH*-wildtype GBMs (**e**). All cytobands shown met the criterion of false discovery rate (FDR) ≤0.25. The annotated cytobands met the criterion of FDR ≤0.05

As expected based on our case selection, Genomic Identification of Significant Targets In Cancer (GISTIC) analysis showed high levels of amplification of 12q14.1 (a region containing *CDK4*) in all gliomas with poor prognosis (i.e., groups 2, 3, 4, and 5) but not in group 1. Similarly, 9p21.3 (a region containing *CDKN2A*) showed frequent deletions in groups 2, 3, 4, and 5 but not in group 1. *IDH*-wildtype tumors had consistent amplifications of 7p11.2 (containing EGFR) and 1q32.1 and deletions of 1p32.3, but only *IDH*-wildtype GBM had consistent deletions at 10q23.31. Interestingly, *IDH*-mutant GBM and *IDH*-mutant LGGs with *CDK4* amplification/*CDKN2A/B* deletion both had amplifications at 2p24.3 (a chromosomal region containing *MYCN*). This was not identified in *IDH*-mutant LGGs with a good clinical outcome or in the *IDH*-wildtype tumors. Group 1 *IDH*-mutant LGGs had significant consistent amplifications at 3p25.2, 5q31.1, 8q24.13, 11q24.2, 13q34, 19q13.12, Xp22.32, and Xq28, as well as consistent deletions at 3p14.1, 9p24.2, 11p12, 13q14.3, 14q24.3, and Xq21.1 that were not identified in any other tumor group (Fig. 7). All cytobands shown met the criterion of false discovery rate (FDR) ≤0.25. The annotated cytobands met the criterion of FDR ≤0.05.

Amplifications and deletions in specific genes of interest were rare in the group 1 *IDH*-mutant LGGs, per our study design (Additional file 1: Figure S1). *IDH*-mutant astrocytomas with poor clinical outcomes (groups 2 and 3) also showed more frequent amplifications of *GLI1*, *KIT*, *KDR*, *MYC*, *MYCN*, *GATA3*, *CCND2*, and *KRAS* as well as more frequent deletions of *PTEN*, *PTPRD*, *ATRX*, and *RB1* (Additional file 2: Figure S2 and Additional file 3: Figure S3).

*IDH*-wildtype groups frequently had amplifications in *EGFR*, *PDGFRA*, *CDK4*, *MDM2*, *MDM4*, *KIT*, and *KDR*, as well as deletions in *CDKN2A/B*, and *PTEN*. *CDK4* amplification and *CDKN2A/B* deletion appear to be almost mutually exclusive, as they only occur together in one *IDH*-wildtype LGG case and one *IDH*-wildtype GBM case (2.3% of cases with these alterations) (Additional file 4: Figure S4 and Additional file 5: Figure S5).

### Analysis of chromothripsis

**Fig. 8:**
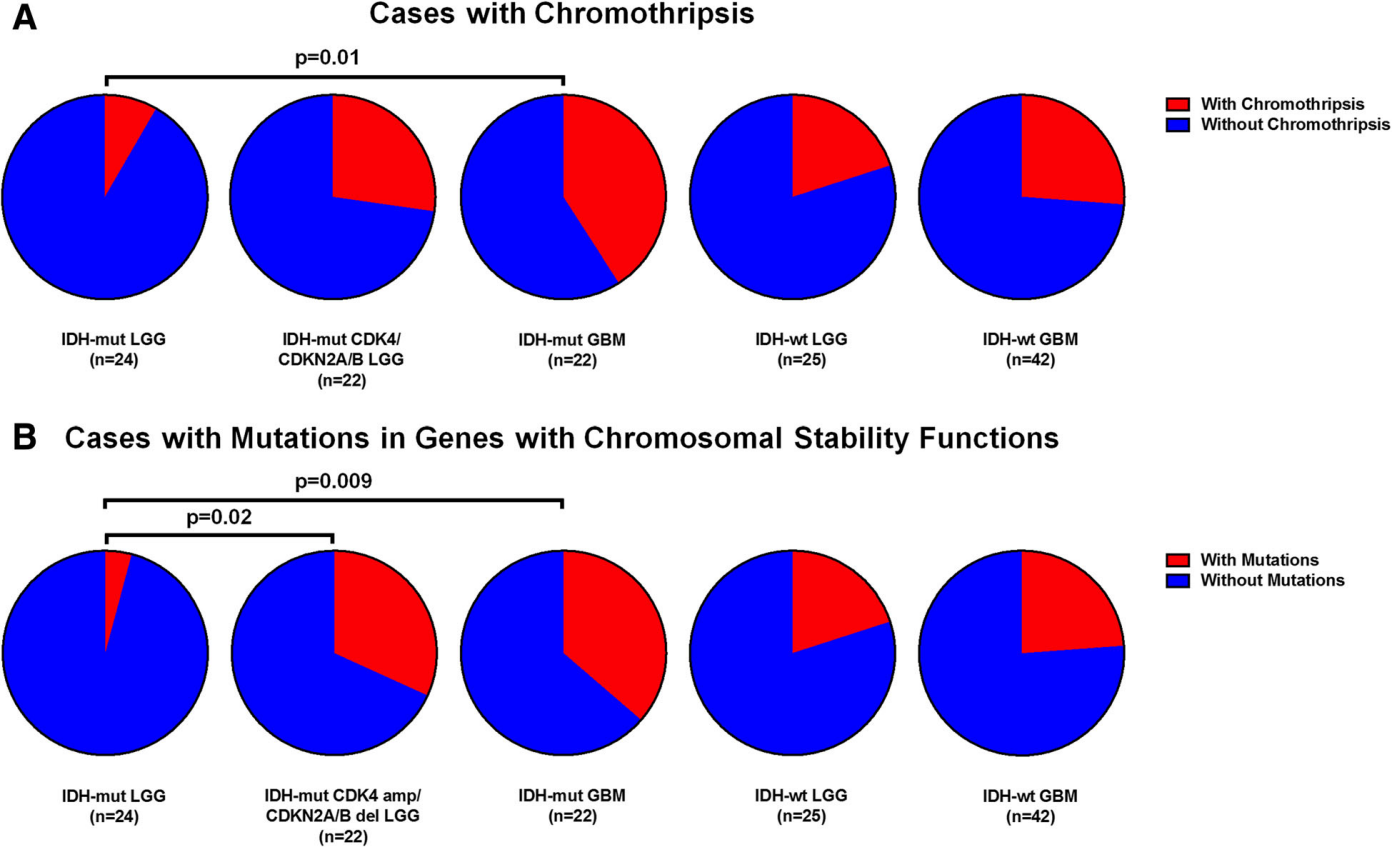
Pie charts illustrating (**a**) the relative frequency of cases with chromothripsis in all 5 astrocytoma subgroups, showing a statistically significant difference between *IDH*-mut LGGs without *CDK4* amplification or *CDKN2A/B* deletion and *IDH*-mut GBMs (*p* = 0.0132) and between *IDH*-mut LGGs without *CDK4* amplification or *CDKN2A/B* deletion and all *IDH*-mut tumors with poor clinical outcome (groups 2 + 3; *p* = 0.0211). Pie charts illustrating (**b**) the relative frequency of cases with mutations involving genes related to preservation of overall chromosomal stability in all 5 astrocytoma subgroups, showing a statistically significant difference between *IDH*-mut LGGs without *CDK4* amplification or *CDKN2A/B* deletion and LGGs with those molecular alterations (*p* = 0.0197) and between *IDH*-mut LGG without *CDK4* amplification or *CDKN2A/B* deletion and *IDH*-mut GBMs (*p* = 0.0086)

Chromothripsis, defined here as 10 or more alternating bands of amplifications and deletions in a single chromosome [9, 21], was identified in at least one tumor in each of the 5 groups analyzed (Table 1). Comparing individual groups, there was a significant difference in the number of cases with chromothripsis between group 1 LGGs without *CDK4* amplification or *CDKN2A/B* deletion and group 3 *IDH*-mutant glioblastomas (*p* = 0.0132) and a significant difference in group 1 LGGs compared to all *IDH*-mutant tumors with poor prognosis (groups 2 and 3 combined) (*p* = 0.0211). No significant difference was observed between groups 2 and 3 (*p* = 0.3475) or between the *IDH*-wildtype groups 4 and 5 (*p* = 0.7681) (Fig. 8a).

### Mutation analysis

Overall mutation load did not differ significantly between any of the tumor groups analyzed (group 1 vs group 2, *p* = 0.3863; group 1 vs group 3, *p* = 0.2745; group 2 vs group 3, *p* = 0.2728; group 3 vs group 5, *p* = 0.3318; or group 4 vs group 5, *p* = 0.5627) (Fig. 3b, d).

Analysis of individual genes in the *IDH*-mutant groups reveals consistently high rates of *TP53* mutations in all 3 groups (91–100% of cases) and relatively high rates of *ATRX* mutations (68–77% of cases). There are other scattered pathogenic mutations, with elevated numbers of *EGFR* (14%) and *PIK3R1* (27%) mutations in the *IDH*-mutant GBM group (Additional file 1: Figure S1, Additional file 2: Figure S2 and Additional file 3: Figure S3).

The *IDH*-wildtype tumor groups have significantly lower rates of *ATRX* mutation in both the LGG group (4%) and GBM group (0%), as well as lower rates of *TP53* mutations in the LGG group (20%) and GBM group (33%). Mutations in *EGFR* (32% in LGG; 24% in GBM), *PTEN* (28% in LGG; 31% in GBM), *NF1* (32% in LGG; 7% in GBM), and *RB1* (12% in LGG; 12% in GBM) were seen significantly more frequently in these tumors than in the *IDH*-mutant groups 1–3 (Additional file 4: Figure S4 and Additional file 5: Figure S5).

### Mutation analysis of genes associated with overall genomic instability

Using a 43-gene panel of genes known to be associated with chromosomal instability (excluding *TP53* due to its relative frequency across all groups), we detected a significant difference in the number of mutations between group 1 *IDH*-mutant LGGs without *CDK4* amplifications or *CDKN2A/B* deletions and group 2 *IDH*-mutant LGGs with either alteration (*p* = 0.0197) as well as between group 1 *IDH*-mutant LGGs and group 3 *IDH*-mutant GBMs (*p* = 0.0086) (Fig. 8b). No significant difference was identified between the two groups of *IDH*-wildtype astrocytomas (*p* = 0.5443). No significant difference was identified between *IDH*-mutant tumors with poor outcomes (group 2 + 3) and *IDH*-wildtype tumors with poor prognosis (group 4 + 5) (*p* = 0.1297), although there was a trend toward fewer mutations in genes specifically associated with chromosomal instability in the *IDH*-wildtype groups (Tables 1 and 2). These data mirror the trend in level of total CNV and chromothripsis identified in each tumor group.

**Table 2:** Summary of mutations in genes with known functions related to maintaining DNA and chromosomal stability for each group

Group	Tumor Type	Mutations in genes with functions related to maintaining
		overall genome/chromosomal stability
1	*IDH*-mut LGG	*BRCA2*
2	*IDH*-mut *CDK4*/	
	*CDKN2A/*B LGG	*APC*, *ATM*, *FANCB*, *FANCD2*, *RAD51* (2), *TOP1*
3	*IDH*-mut GBM	*APC* (4), *BLM*, *BRCA2*, *SMC1* (2)
4	*IDH*-wt LGG	*BLM*, *FANCB* (2), *FANCE*, *LIG4*
5	*IDH*-wt GBM	*ATR, BRCA2* (2), *CLSPN, FANCI* (2), *FANCM* (2), *PRKDC, REV3*

## Discussion

Diffuse gliomas represent approximately 27% of all primary brain tumors and approximately 81% of all malignant brain tumors [29, 30], making them an intense subject of study and public health expenditure. The recent changes to glioma classification in the 2016 WHO classification system are based around the beneficial role of *IDH*-mutation in gliomas [25]; however, significant molecular heterogeneity exists within the lower-grade*IDH*-mutant and wildtype gliomas. More work is necessary to further stratify *IDH*-mutant astrocytomas [44], and there is evidence that many *IDH1/2*-wildtype LGGs may be biologically identical to *IDH1/2*-wildtype glioblastomas [17, 34]. In addition, new methods to analyze whole genome genetic and epigenetic signatures are leading to new definitions for many of these tumor groups with significant prognostic implications [4, 38, 43].

We previously reported that increased CNV is associated with a more aggressive biological behavior and poor overall survival in *IDH*-mutant LGGs [36, 37]. With whole genome analysis in the current study, we show that CNV correlates with clinical outcome, and was significantly lower in the *IDH*-mutant LGGs compared to the *IDH*-mutant LGGs with *CDK4* or *CDKN2A/B* alterations or *IDH*-mutant GBMs. (Figs. 3a and 4). These results confirm our previous findings, in which *IDH*-mutant LGG cases selected solely on the basis of poor clinical outcome displayed significantly higher levels of CNV before progression to GBM than a cohort with more conventional progression-free and overall survival [36]. The elevated CNV levels in *IDH*-mutant LGGs with *CDK4* or *CDKN2A/B* alterations and *IDH*-mutant GBM represent a heterogenous assortment of genomic alterations within the *IDH*-mutant group with only a few consistent areas of gains and losses (Fig. 5b-c) whereas a large fraction of the CNV in *IDH*-wildtype tumors arose from consistent amplifications in chromosome 7p (containing *EGFR*), and deletions in chromosomes 9p and 10 (Fig. 6).

Although the overall CNV changes seem to occur before histologic progression to GBM in cases with other negative prognostic factors and/or clinically demonstrated poor outcomes, there is still uncertainty in the exact connection to elevated levels of CNV and the driving force behind this poor progression. Our data also agrees with the previously demonstrated data that *CDK4* and *CDKN2A/B* alterations are prognostic factors within the *IDH*-mutant LGGs [44]. While worse prognosis seems to correlate with *CDK4* or *CDKN2A/B* status, our earlier study [36] showed only a fraction of the rapidly progressing tumors had these specific alterations, yet all of them had high overall CNV, indicating that it may be an earlier event or a separate phenomenon altogether. Further analysis of CNV data may help determine if the *IDH*-mutant LGGs with *CDK4* and/or *CDKN2A/B*alterations are actually early GBMs or simply under-sampled tumors, similar to current thinking on many *IDH*-wildtype LGGs [3, 42]. While it is reasonable to argue that our cohort of *IDH*-mutant LGGs without *CDK4* or *CDKN2A/B* alterations show low CNV because they selectively exclude tumors with specific known amplifications/deletions to enrich the other cohorts, if this were to hold true, the clinical outcome would likely also follow the same pattern and would show worse outcome within the other groups containing *CDK4* amplification or *CDKN2A/B* deletion. *CDK4* and *CDKN2A/B* did not show a prognostic difference in *IDH*-mutant GBMs or *IDH*-wildtype LGGs or GBMs, and the overall CNV was not different between these two groups (Fig. 2a-c), so the effect of both of these alterations seems limited to *IDH*-mutant LGG cases. *CDK4* amplification and *CDKN2A/B* deletion also appear to be mutually exclusive, with only two total cases (2.3%) having both molecular alterations (Additional file 4: Figure S4 and Additional file 5: Figure S5).

An additional finding in these tumor groups is the trend toward more frequent mutations in genes associated with overall chromosomal stability in groups with worse clinical outcomes (groups 2–5) compared to the group with relatively favorable outcomes (group 1) (Fig. 8b, Table 2). This correlates positively with the trends toward increased CNV levels and number of cases with chromothripsis and inversely with the progression-free and overall survival in these groups (Table 1). The number of mutations in genes with chromosomal stability functions and cases with chromothripsis are somewhat lower in the *IDH*-wildtype cohorts compared to groups 2 and 3 in the *IDH*-mutant cohorts, despite having statistically identical CNV levels (Fig. 8). This difference may be explained by the fact that a large portion of the CNV in these *IDH*-wildtype groups is more homogeneously associated with specific chromosomal regions (7, 9p, 10) instead of more diffusely distributed as seen in the *IDH*-mutant groups with high CNV and poor outcome (Figs. 5 and 6).

This process also provides a potential mechanistic explanation for the widespread genomic alterations and the worse prognosis associated with this increase in CNV in at least a subset of cases. Inactivating mutations in genes associated with maintenance of genetic and chromosomal integrity, and the resulting increase in CNV, allows for rapid and widespread changes to the genome, including chromothripsis, and has the potential to cause more frequent gains of oncogenes and loss of tumor suppressor genes and drive tumor formation and progression towards malignancy [11, 19, 20, 41, 46]. This may also suggest a different molecular mechanism underlying total CNV levels in *IDH*-mutant and *IDH*-wildtype groups. At this point, however, we can only state that these factors are all correlated with poor clinical outcome, but no causative links can definitively be made.

The present study reinforces our previous findings [36, 37] demonstrating that elevated CNV is associated with poor outcome in grade II and III *IDH*-mutant astrocytomas, and presents this as a potential prognostic factor. We demonstrate for the first time that higher CNV is associated with previously established prognostic factors within the *IDH*-mutant LGG subgroup, such as *CDK4* amplification and *CDKN2A/B* deletion. This study is also the first to demonstrate a significant quantitative difference in mutations of genes related to chromosomal stability in groups with higher CNV and worse clinical outcomes (Fig. 8b).

It is important to note that while many of the genetic and epigenetic methods used to generate these data are currently only used for research purposes, recent proof-of-concept studies have demonstrated that specific and large-scale genetic and epigenetic alterations can be identified rapidly and relatively inexpensively [12, 18], including overall methylation patterns indicative of *IDH1/2* status, methylation of key gene promotors, CNV, mutations, and gains and losses of key genes and chromosomal regions. These studies have demonstrated that with newer techniques these molecular factors can be identified in approximately the time that it takes to make a histologic diagnosis. It is therefore conceivable that CNV and other molecular factors identified in this report could soon be used clinically at the time of initial diagnosis to help guide prognosis and treatment strategies.

## Conclusions

Our results support our previous findings that *IDH*-mutant lower-grade astrocytomas with higher total CNV are associated with poor clinical outcome and behave more consistently with *IDH*-mutant GBM than other *IDH*-mutant LGGs with low CNV, and suggest that CNV could be a viable prognostic factor in these tumors alongside *IDH1/2* mutations, *CDK4* amplifications, and *CDKN2A/B* deletions. We demonstrated that high CNV occurs in *IDH1/2*-wildtype astrocytomas and glioblastomas which also have poor prognoses, although the reason underlying elevated CNV may be different in *IDH*-mutant and *IDH*-wildtype tumors. We also provide a possible mechanism for the overall CNV differences in these astrocytoma subgroups, as the CNV levels seem to correlate with numbers of mutations in genes with roles in maintaining genomic stability. These results suggest that high overall CNV negate the beneficial effects of *IDH1/2* mutation, and could potentially be used as a prognostic marker in *IDH*-mutant astrocytomas in the future.

## Additional files

Additional file 1: **Figure S1.** Summary plot showing the frequency of genes with pathologic mutations and amplifications, *IDH*-mutant LGGs without *CDK4* amplification or *CDKN2A/B* deletion. (TIF 91 kb)

Additional file 2: **Figure S2.** Summary plot showing the frequency of genes with pathologic mutations and amplifications/deletions, *IDH*-mutant LGGs with either *CDK4* amplification or *CDKN2A/B* deletion. (TIF 90 kb)

Additional file 3: **Figure S3.**. Summary plot showing the frequency of genes with pathologic mutations and amplifications/deletions, *IDH*-mutant GBMs. (TIF 86 kb)

Additional file 4: **Figure S4.** Summary plot showing the frequency of genes with pathologic mutations and amplifications/deletions, *IDH*-wildtype LGGs. (TIF 95 kb)

Additional file 5: **Figure S5.** Summary plot showing the frequency of genes with pathologic mutations and amplifications/deletions, *IDH*-wildtype GBMs. (TIF 140 kb)

## Data Availability

The full dataset used in this study is freely available at www.cbioportal.org and https://www.cancer.gov/about-nci/organization/ccg/research/structural-genomics/tcga.
