# Supplementary figures and images for: Total copy number variation as a prognostic factor in adult astrocytoma subtypes
Source: Acta Neuropathol Commun. 2019 Jun 10;7:8. doi: 10.1186/s40478-019-0746-y (PMC6556960; doi:10.1186/s40478-019-0746-y)

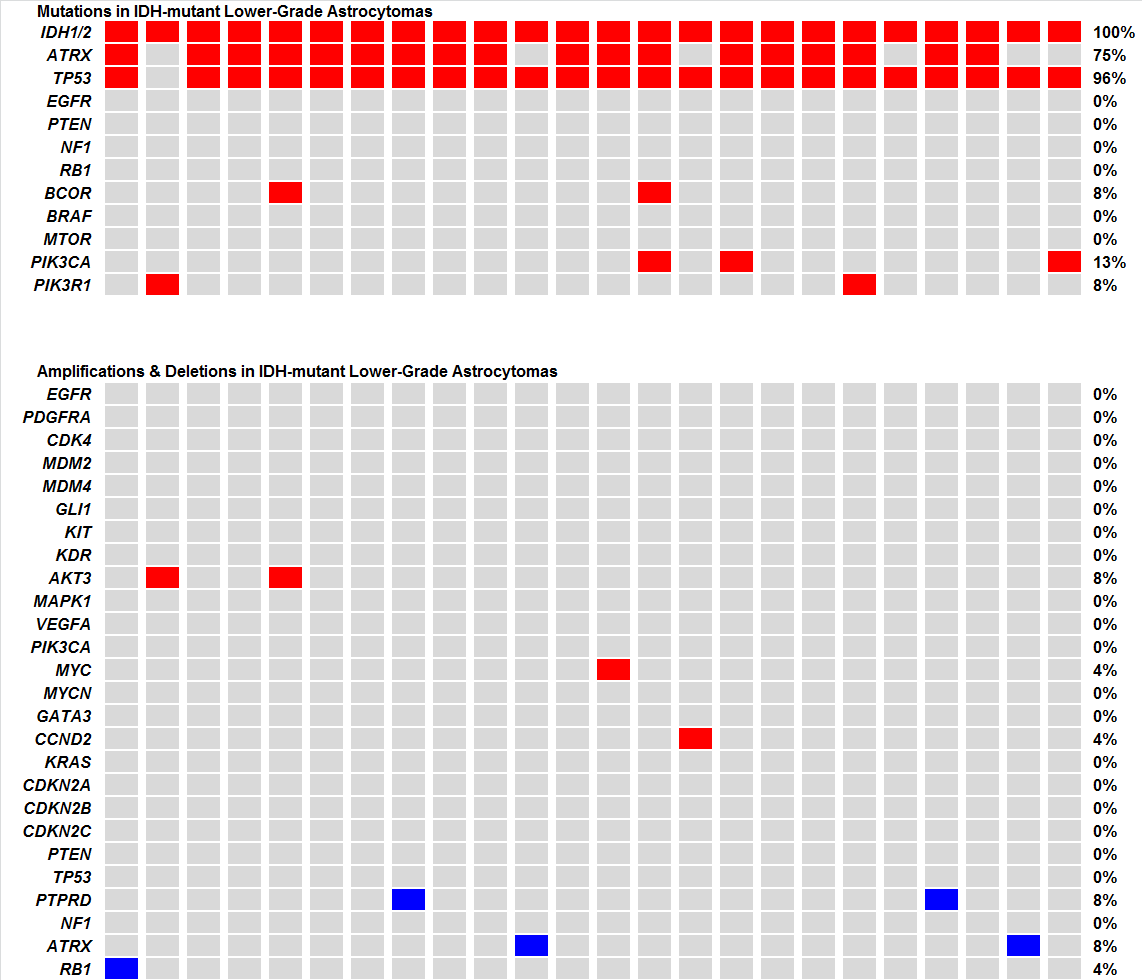

Supplement: Supplementary file 1 — Figure S1. Summary plot showing the frequency of genes with pathologic mutations and amplifications, IDH-mutant LGGs without CDK4 amplification or CDKN2A/B deletion. (TIF 91 kb) [file 40478_2019_746_MOESM1_ESM.tif]

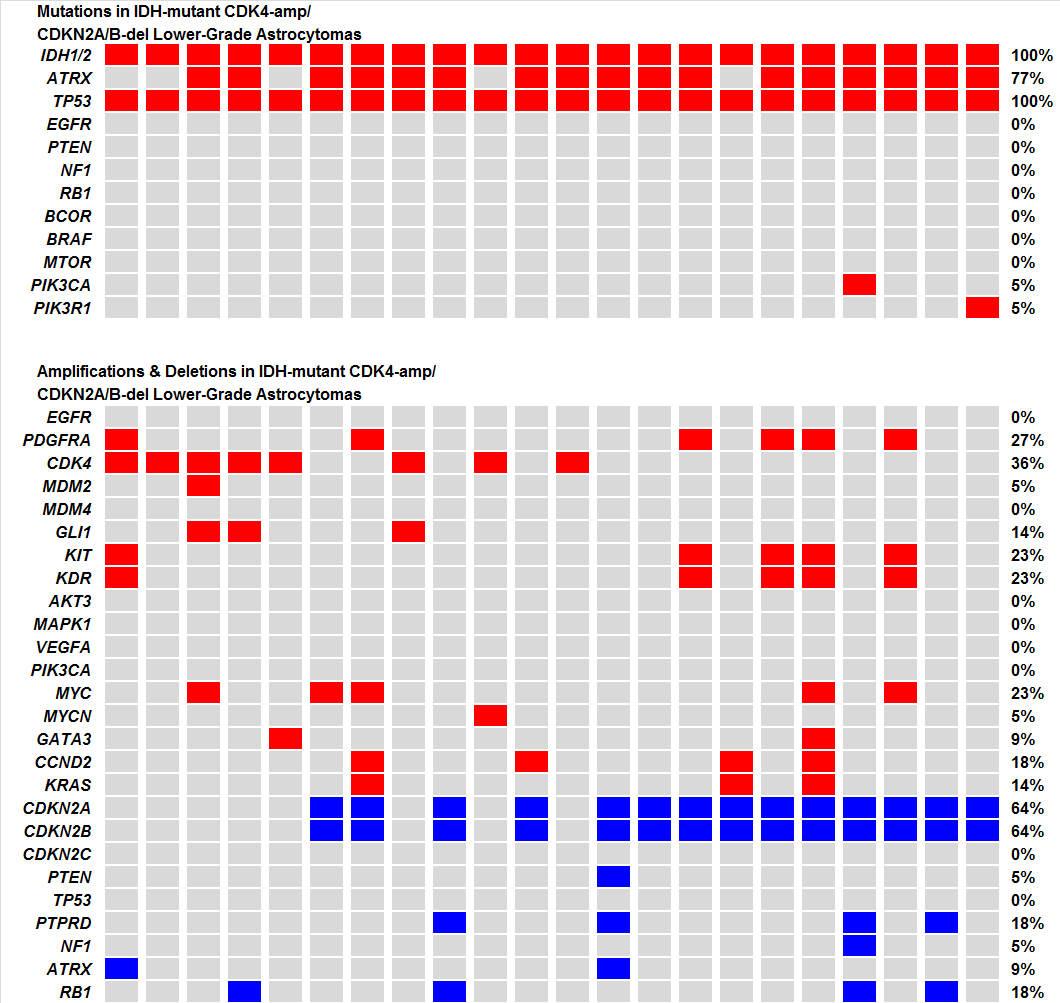

Supplement: Supplementary file 2 — Figure S2. Summary plot showing the frequency of genes with pathologic mutations and amplifications/deletions, IDH-mutant LGGs with either CDK4 amplification or CDKN2A/B deletion. (TIF 90 kb) [file 40478_2019_746_MOESM2_ESM.tif]

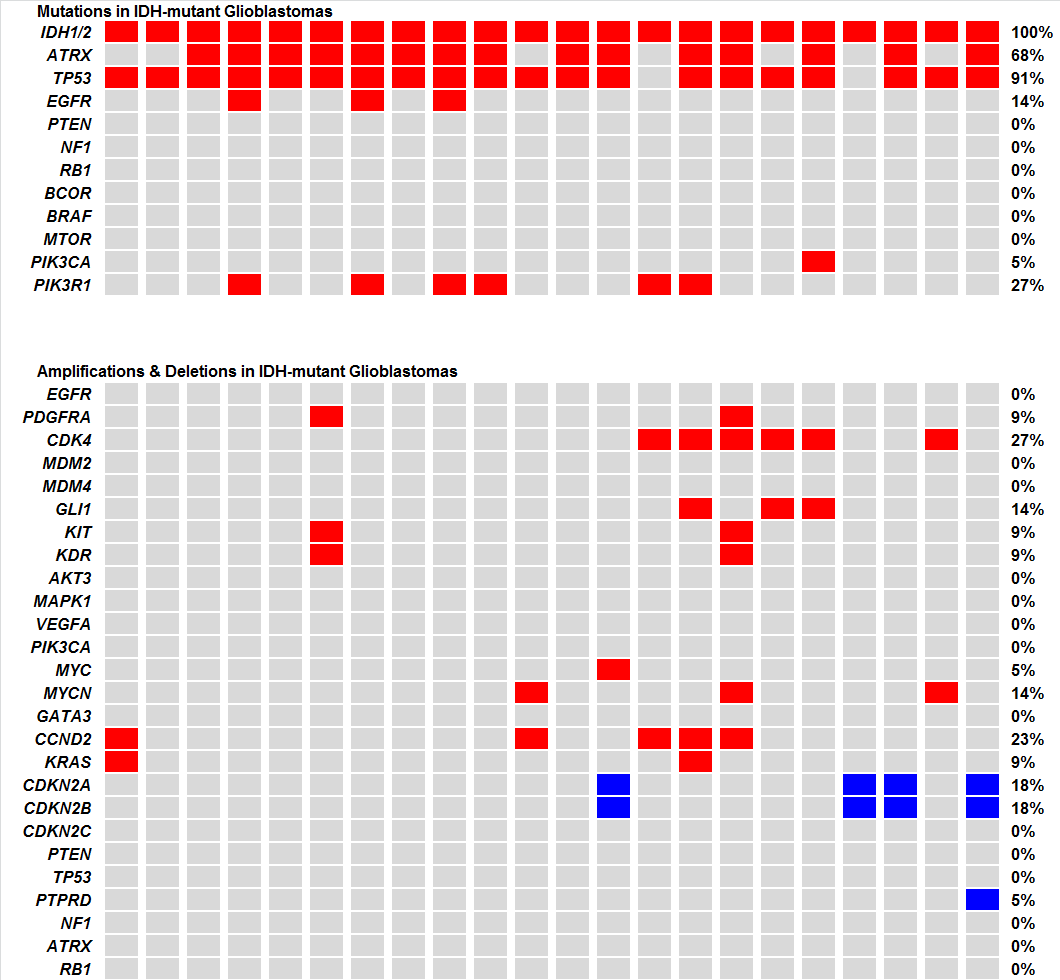

Supplement: Supplementary file 3 — Figure S3.. Summary plot showing the frequency of genes with pathologic mutations and amplifications/deletions, IDH-mutant GBMs. (TIF 86 kb) [file 40478_2019_746_MOESM3_ESM.tif]

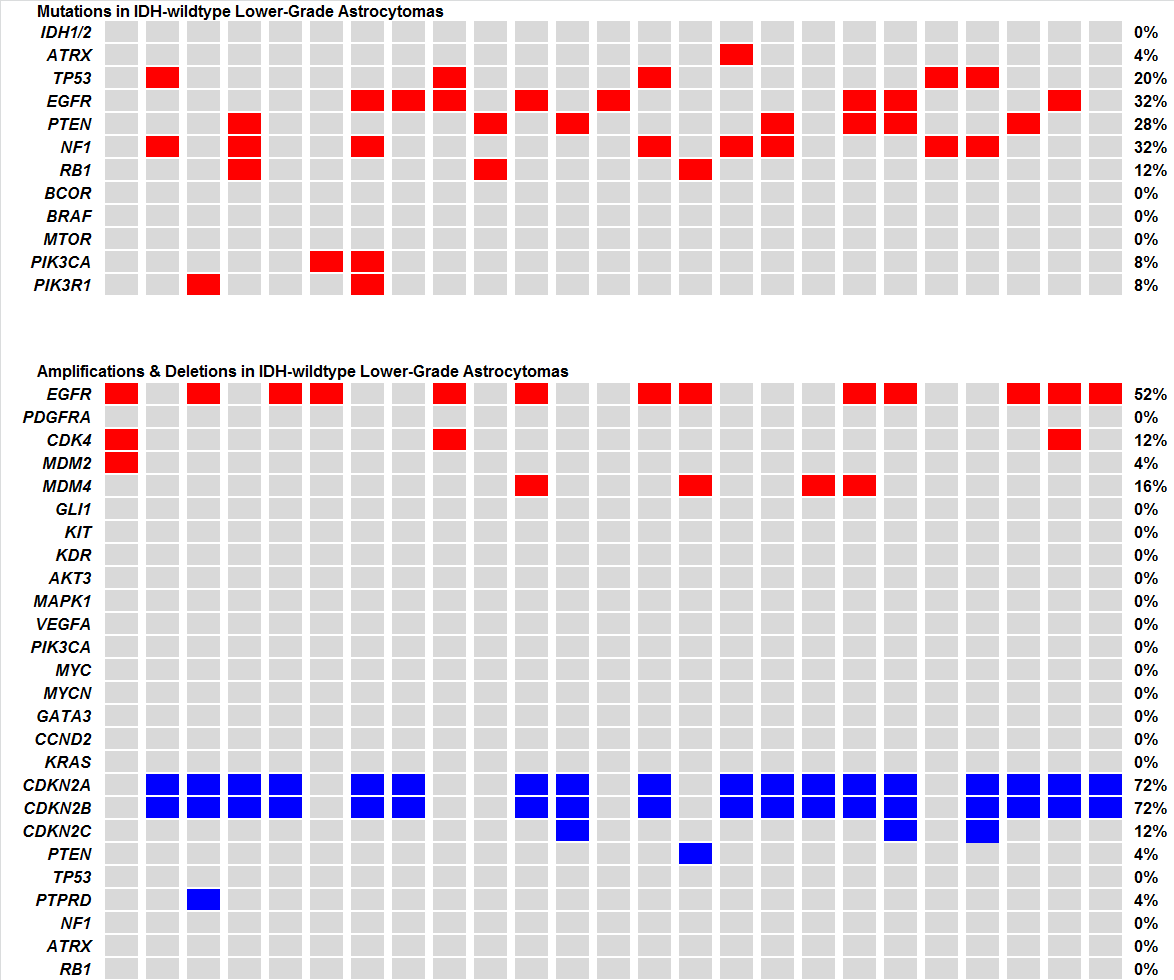

Supplement: Supplementary file 4 — Figure S4. Summary plot showing the frequency of genes with pathologic mutations and amplifications/deletions, IDH-wildtype LGGs. (TIF 95 kb) [file 40478_2019_746_MOESM4_ESM.tif]

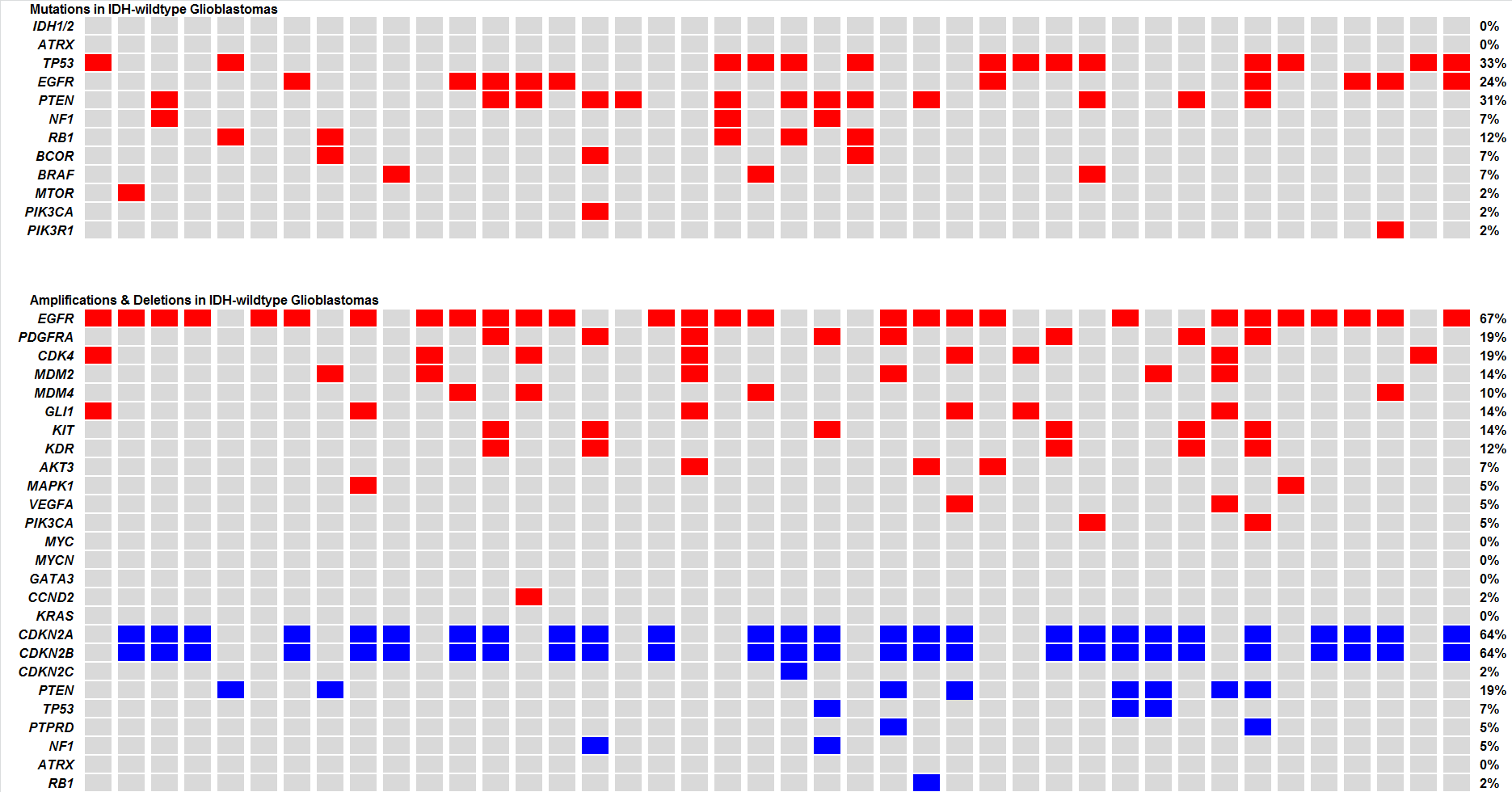

Supplement: Supplementary file 5 — Figure S5. Summary plot showing the frequency of genes with pathologic mutations and amplifications/deletions, IDH-wildtype GBMs. (TIF 140 kb) [file 40478_2019_746_MOESM5_ESM.tif]
